# P-134. Sarcina ventriculi, pathogen or commensal? Case series and review of the literature

**DOI:** 10.1093/ofid/ofae631.339

**Published:** 2025-01-29

**Authors:** Zaid Al khouri, Anood Al Qura'an, Satya Koppada, Maryam Mubashir, Hassaan Zia, Paulette Pinargote, Wilmer Salazar

**Affiliations:** Louisiana State University Health Sciences Center Shreveport, Shreveport, Louisiana; Louisiana State University Health Sciences Center Shreveport, Shreveport, Louisiana; LSU Health Science Center Shreveport, Shreveport, Louisiana; Louisiana State University Health Sciences Center Shreveport, Shreveport, Louisiana; Louisiana State University Health Sciences Center Shreveport, Shreveport, Louisiana; Louisiana State University Health Services Center Shreveport, shreveport, Louisiana; Universidad Catolica Santiago de Guayaquil, Shreveport, Louisiana

## Abstract

**Background:**

Sarcina, or Clostridium, ventriculi (SV), is a non-motile, gram-positive coccus with anaerobic fermentative properties. Due to its rarity, comprehensive knowledge about it is lacking. This study aims to elucidate its epidemiology and common manifestations.

**Figure:**
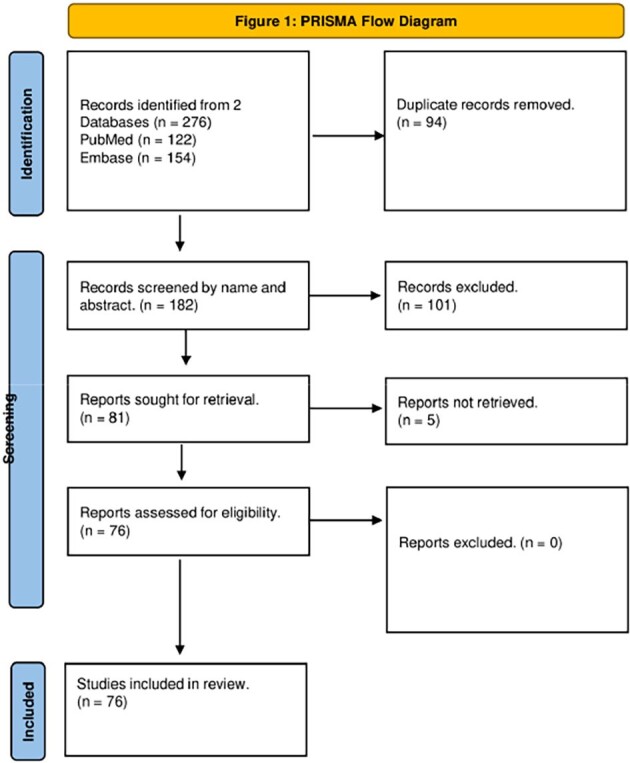

**Methods:**

We searched PUBMED and EMBASE utilizing terms as 'Sarcina', 'Ventriculi', and 'Clostridium' with no restrictions. Figure 1 contains the PRISMA diagram for case selection.

**Results:**

We identified 88 cases and included 2 cases from our institution. The mean age was 48 ranging from 10 months to 89 years with an equal distribution between genders. 21% had Diabetes mellitus type 2 and 50% had baseline GI comorbidity primarily affecting the esophagus or stomach in 27% respectively. 37% were concurrently on antacids and prokinetics. The most common symptoms were abdominal pain (58%), nausea/vomiting (41%), anorexia/weight loss (18%), and diarrhea (12%).  Pertinent labs were anemia, leukocytosis, and elevated inflammatory markers. Mural inflammation, ulcers, retained food with distension, pneumatosis, perforation, necrosis, masses, or obstruction were common on radiological and endoscopic workup. Organism was mostly isolated from gastric (73%) and esophageal (18%) tissue using endoscopic and/or surgical procedures (76 and 27%, respectively). It was also isolated from respiratory tissue (3%), anaerobic blood culture (3%), lymph nodes (2%), urine (1%), and cerebral ventricles (1%). Co-infection with candida in 8% and H. Pylori in 4% was noted. 57% received antibiotics, being combination therapy with metronidazole and fluroquinolones, the most therapy used.

77% had successful clinical recovery, 11% expired; the outcome for 12% of patients remains unknown. Antibiotic therapy was used in 67% and 40% of patients who clinically recovered and expired respectively.

**Conclusion:**

In most cases, SV is a true pathogen responsible for symptoms rather than a commensal; however, larger-scale studies are needed to discern pathophysiology. Most cases present with an underlying GI comorbidity including dysmotility which might predispose to colonization. Metronidazole and fluroquinolones provide optimal clinical response.

**Disclosures:**

**All Authors**: No reported disclosures

